# Epidemiology and control strategies for foot-and-mouth disease in livestock and wildlife in Uganda: systematic review

**DOI:** 10.1007/s11259-025-10791-z

**Published:** 2025-06-16

**Authors:** Benedicto Byamukama, Asfor Amin, Frank Nobert Mwiine, Abel Bulamu Ekiri

**Affiliations:** 1 Department of Comparative Biomedical Sciences, School of Veterinary Medicine, Faculty of Health and Medical Sciences, University of Surrey, Daphene Jackson Road, Guildford, GU2 7AL UK; 2 School of Biosecurity, Biotechnical and Laboratory Sciences, College of Veterinary Medicine Animal Resources and Biosecurity, Makerere University, Kampala, Uganda

**Keywords:** Foot-and-mouth disease, Virus, Cattle, Epidemiology, Challenges, Uganda

## Abstract

Foot-and-mouth disease (FMD) remains a persistent threat to the livestock sector in Uganda and globally. To understand the reasons for continued FMD outbreaks in Uganda, a systematic review was conducted to examine FMD epidemiology, control strategies, and related challenges. Literature searches were performed in PubMed, ScienceDirect, Scopus, and Web of Science databases for the period 1958–2022, following the Preferred Reporting Items for Systematic Reviews and Meta-Analysis guidelines. Additionally, FMD reports for the same period were retrieved from the World Reference Laboratories for FMD and the World Organization for Animal Health/Food and Agriculture Organization FMD Laboratories Network websites. A total of 62 documents (26 studies and 36 reports) were eligible for analysis. Results showed that Uganda experienced 17 to 37 FMD outbreaks annually. Most of the studies focused on cattle (*n* = 20), with few targeting African buffalo (*n* = 3) and small ruminants (*n* = 2). The reports revealed that serotype O was the most isolated, followed by A, SAT 1, SAT 2, and SAT 3. FMD clustering was observed near international borders with Kenya and Tanzania and around Queen Elizabeth National Park. Identified risk factors included dry season, animal movements, proximity to borders, and pastoralism. The key control challenges included limited diagnostic capacity, FMD virus diversity, vaccine mismatch, and low vaccination coverage. The findings highlight the need for enhanced FMD monitoring, surveillance, and control strategies that consider circulating serotypes and topotypes, and improvement in vaccination coverage and strengthening of diagnostic capacity. Further research is needed to address the epidemiology of FMD in understudied species, including small ruminants, swine, and wildlife.

**Supplementary Information:**

The online version contains supplementary material available at 10.1007/s11259-025-10791-z.

## Introduction

Foot-and-mouth disease (FMD) is a highly contagious viral disease that affects cloven-hooved animals, including cows, sheep, pigs, goats, and a variety of wild animal species (Donaldson 1987). The global economic impact of FMD is substantial, with estimates ranging from $6.5 to 21 billion annually (Knight-Jones and Rushton 2013). This impact is particularly pronounced in regions where the disease is endemic, such as Africa, Asia, and the Middle East (OIE/WOAH 2021). In Africa alone, FMD is estimated to affect more than 50 million animals each year and cost the continent an estimated $830 million (Rushton and Knight-Jones 2015). In Uganda, FMD is endemic with outbreaks reported annually throughout the country (Ayebazibwe et al. 2010c; Namatovu et al. 2015a, b; Mwiine et al. 2019; Mugezi et al. 2020; Okello et al. 2022), resulting in significant production losses for livestock farmers (Baluka 2016), and impacting livestock trade (Kerfua et al. 2023).

The typical clinical signs of FMD include the development of painful blisters on the mouth and feet of infected animals (Donaldson 1987). The blisters eventually rupture, leaving painful lesions (Kitching and Hughes 2002). The lesions can have a profound impact on the general health and welfare of affected animals, including interference with the ability to eat as well as inducing lameness which compromises mobility of the animals (Kitching and Hughes 2002). In addition, infected animals may exhibit fever, contributing to distress and discomfort. The FMD virus is shed in significant amounts from ruptured vesicles and lesions but is also present in all secretions and excretions of sick animals. The virus is spread through contact with infected animals, aerosols, and ingestion of infected materials (Alexandersen et al. 2003; Sarry et al. 2022).

Foot-and-mouth disease viruses (FMDV) have been classified into seven regional pools through genetic and antigenic analyses (FAO/OIE 2012). The regional pools are designated as: Pool 1 (East Asia), Pool 2 (South Asia), Pool 3 (West Eurasia), Pool 4 (East Africa), Pool 5 (West Africa), Pool 6 (South Africa), and Pool 7 (South America). Within each of the distinct FMDV genetic groupings, FMDV tend to persist and re-emerge, creating cycles of transmission that can affect multiple neighboring countries within the same region (Knowles and Samuel 2003; Rweyemamu et al. 2014). The regional distribution of genotypes serves as a basis for the global FMD control strategy, as outlined in the roadmap jointly developed by the World Organization for Animal Health (WOAH) and the Food and Agriculture Organization (FAO) in 2012 (Sumption et al. 2012). The strategy aims to understand the epidemiology of FMDV and to develop effective control strategies on a global scale.

Several studies have investigated the factors that contribute to the occurrence of FMD across the world. Animal movement is an important risk factor for the spread of FMD within endemic regions. In Uruguay, animal movements, combined livestock with crop production, large and medium size farms, geographical location, type of livestock kept, and delay in the detection of the index cases were associated with FMD outbreaks (Iriarte et al. 2023). In the UK, larger cattle herds and large farms were associated with the 2001 FMD outbreak (Ferguson et al. 2001). The structure and density of the livestock production sector were found to play a pivotal role in the occurrence of FMD during the 2001 epidemic in the Netherlands (Bouma et al. 2003), the 2010 epidemic in Japan (Muroga et al. 2013), and the 1997 epidemic in Taiwan (Yang et al. 1999). Other factors were related to indirect transmission of FMD, including inadequate biosecurity measures (Ellis-Iversen et al. 2011), and the movement of people, vehicles, and farm equipment from infected areas (Muroga et al. 2013). In African countries, higher pig density, distance from international borders, and proximity to wildlife-protected areas (Allepuz et al. 2015; Abdela 2017; Chimera et al. 2022), have been associated with FMD occurrence. In Uganda, the factors linked to the persistent occurrence of FMD include livestock movements, seasonal changes, and high livestock density (Munsey et al. 2021; Okello et al. 2022).

There are ongoing efforts to control FMD at the global, regional, and national levels. The global FMD control strategy aligns with the Progressive Control Pathway for FMD (PCP-FMD), a comprehensive framework developed by the FAO and the WOAH. It encompasses stages ranging from identifying national FMD risk and control options to achieving the status of freedom without vaccination. The global strategy emphasizes timely disease detection, robust surveillance, established vaccination programs, strict movement control, capacity building, international collaboration, and stakeholder engagement (Sumption et al. 2012). By following the PCP-FMD and tailoring efforts to specific national contexts, countries work towards the goal of achieving freedom from FMD without vaccination status, while addressing the unique challenges at the country level (Sumption et al. 2012).

In Uganda, the current officially recognized status of WOAH is stage 2 of the PCP-FMD (FAO 2024). For Uganda to progress from stage 2 (characterized by a reduction in FMD impact in targeted sectors/areas) to stage 3 (characterized by a reduction in FMD virus circulation), several key tasks must be completed as recommended by the PCP-FMD roadmap. The key tasks include: enhancing surveillance systems for prompt and accurate detection and reporting of outbreaks; increasing vaccination coverage of susceptible animals using vaccines that match circulating serotypes; implementing risk-based zoning and compartmentalization to control animal movements between areas of varying FMD risk; improving biosecurity and hygiene practices on farms, at markets, and in slaughterhouses; strengthening the capacity of veterinary services for effective FMD management; community engagement and awareness programs to encourage participation in FMD control activities and surveillance; supporting research on FMD epidemiology, vaccine development, and diagnostic tools; and understanding the role of wildlife in FMD transmission (FAO/OIE 2012a). Implementing the above tasks could help control FMD and reduce its impact on the livestock sector in Uganda.

In Uganda, despite the implementation of some control strategies, the country has continued to experience annual FMD outbreaks in several areas (Namatovu et al. 2015a, b; Kerfua et al. 2018; Mwiine et al. 2019), especially along the cattle corridor region, where cattle rearing is concentrated, along with other FMD susceptible livestock species including sheep, goats and pigs. The main control strategies for FMD in Uganda are ring vaccinations and restricting animal movements in affected areas (Ayebazibwe et al. 2010c; Muleme et al. 2012). The government enforces quarantine measures and restricts animal movements in affected areas during FMD outbreaks. (Ayebazibwe et al. 2010c; Muleme et al. 2012). The causes of frequent annual FMD outbreaks in Uganda remain unclear. Investigating FMD occurrence patterns and circulating virus serotypes could provide insights on the outbreaks and inform prevention and control strategies. To address this gap, we conducted this review to examine the existing evidence on FMD occurrence patterns, risk factors, control strategies, and the associated challenges.

## Methods

### Study design

We conducted a systematic review following the Preferred Reporting Items for Systematic Reviews and Meta-Analysis (PRISMA) guidelines (Moola et al. 2020). The specific questions addressed in this review were: shown below.

1. What are the circulating strains of FMDV in livestock and wildlife in Uganda?
2. What are the risk factors for FMD in livestock and wildlife in Uganda?
3. What are the control strategies and related challenges of FMD in Uganda?

### A preliminary search of the research topic

A preliminary search on the topic was conducted in Google Scholar and PubMed to check if a similar study had not been conducted in Uganda with the aim of avoiding duplication. It was also intended to check if a reasonable number of articles were available for review.

### Inclusion and exclusion criteria

Inclusion: All available and accessible original studies in English published in peer-reviewed journals between 01 January 1958 (the earliest available FMD reports from Uganda) and 31 December 2022 were eligible for inclusion. In addition, reports published in the same period by the reference laboratory network (RLN) of the World Reference Laboratories for FMD (WRL-FMD) and the World Organization for Animal Health (WOAH)/FAO reference laboratory network for FMD were included. All studies conducted on FMD susceptible livestock and wildlife species from any part of Uganda were included.

Exclusion: The following studies were excluded: studies reporting on FMD outside Uganda, studies on hand, foot-and-mouth disease, duplicated records, studies without abstracts or without available full texts, and studies focused on case study reports, or description of disease, or diagnosis and vaccine development. Furthermore, conference abstracts, posters, opinion pieces, parliamentary/ministry reports were excluded.

### Search strategy

The search was focused on 4 databases: Scopus, Web of Science, PubMed, and Science Direct databases. Search terms were defined based on the research questions and used to identify relevant studies (Supplementary Table S1). The published reports of the WRL-FMD (including a report by Ferris and Donaldson 1992) were retrieved from the websites of WOAH/FAO (WOAH/FAO 2023) and WRL-FMD (WRL-FMD 2023a).

### Title and abstract screening and data extraction

All identified records in the 4 databases were selected and extracted by exporting either to Excel directly or to EndNote and then generating an Excel file from EndNote. All exported records were then combined into a single Excel file. All records were subjected to title and abstract screening. All duplicates and studies without retrievable abstracts or full articles were excluded.

Following screening and selection of titles and abstracts, full articles for the selected eligible studies were downloaded. Data were extracted from articles and entered in the Excel file. The extracted data included author, article title, study description, study objective, methods used, study design, target population, sample size, study area, study year, key findings of the study, information on FMD occurrence, FMDV serotypes, risk factors, control strategies, control challenges, and study gaps.

### Quality assessment

Quality assessment was performed using the Joanna Briggs Institute (JBI) Critical Appraisal Checklist for Analytical Cross-Sectional Studies (Moola et al. 2020). The checklist evaluated the following questions: whether the study question was clearly stated; whether the inclusion criteria were appropriate for the study question; whether the search strategy was adequate; considered if the sources and resources used for the search were sufficient; and if the criteria for appraising studies were appropriate. The response to each question was either “Yes”, “No”, “Unclear”, or “Not applicable”. Depending on the response, studies were classified as high, moderate, or low quality. High-quality studies were studies with a higher frequency of “Yes” responses and fewer “No,” “Unclear,” or “Not applicable” responses, based on the checklist.

### Data analysis

Data from the reviewed studies were summarised using frequency distribution tables and graphs where appropriate, synthesized and reported.

## Results

The results are presented under the following subsections: literature search and screening results, characteristics of journal articles and reports, frequency of FMD outbreaks in Uganda, prevalence of FMD in Uganda, circulating serotypes and topotypes and their distribution in Uganda, risk factors for FMD in Uganda, FMD control strategies in Uganda and FMD control challenges in Uganda.

### Literature search and screening results

**Fig. 1:**
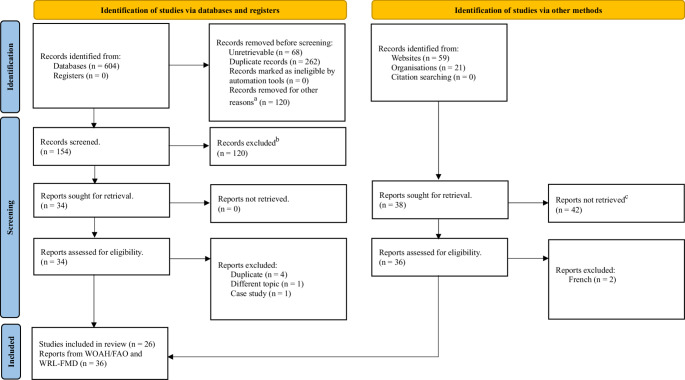
The PRISMA flow diagram for the systematic review showing the number of abstracts screened, the full-text articles retrieved, and the reports retrieved from the WLR-FMD and WOAH/FAO websites (a = article on hand-foot-and-mouth disease, other languages other than English, records from countries other than Uganda. b = Different geographical location, topics other than FMD. c = article focused on other diseases or countries other than Uganda)

Data for this review were extracted from 26 articles, selected following the screening of 604 journal articles as shown in the PRISMA diagram (Fig. 1). Furthermore, 36 of the 80 reports identified on the WOAH/FAO and WRL-FMD websites were included (Fig. 1).

### Characteristics of the journal articles and reports

The characteristics of the reviewed articles are summarised in Table 1, including the author, description of the study, study design, target population, sample size, area of study, and the period during which the study was conducted. The period of publication of the studies ranged from 1990 to 2022 (Supplementary Table S2). The targeted animal species consisted of cattle, African buffaloes (*Syncerus caffer)*, small ruminants (sheep and goats), and, to a limited extent, other species (Impalas, Giraffes, Eland, Hartebeests, and Waterbucks). The studies were carried out in varied geographical areas, including selected districts and regions within Uganda and national parks. The study designs included qualitative risk assessment (*n* = 1), cross-sectional studies (*n* = 17), retrospective (*n* = 4), longitudinal observational (*n* = 1), qualitative and quantitative research (*n* = 1), descriptive and retrospective (*n* = 2).

**Table 1:** Characteristics of the reviewed articles

Author	Study description	Study design	Target population	Sample size	Area of study	Period
Ahmed et al. 2022.	Complete coding genome sequences of five Foot-and-mouth disease viruses belonging to serotype O, isolated from cattle in Uganda in 2015 to 2016.	Cross-sectional	Cattle	5 FMDV serotype O strains.	Four different regions of Uganda: northern, western, eastern and central.	2015 and 2016.
Ayebazibwe et al., 2010c.	Patterns, risk factors and characteristics of reported and perceived Foot-and-mouth disease in Uganda.	Retrospective	Ruminants	311 reported FMD outbreaks in 56 districts.	Various districts in Uganda, analysing FMD outbreaks in different geographical regions.	2001 to 2008.
Ayebazibwe et al., 2010a.	Antibodies Against Foot-and-mouth disease (FMD) virus in African Buffalos (*Syncerus caffer*) in selected national parks in Uganda (2001–2003).	Cross-sectional	African buffalos (*Syncerus caffer*)	38 buffalo samples.	National parks in Uganda, including Lake Mburo, Kidepo Valley, Murchison Falls, and Queen Elizabeth National Parks.	2001 and 2003.
Ayebazibwe et al., 2010b.	The role of African buffalos (*syncerus caffer*) in the maintenance of Foot-and-mouth disease in Uganda.	Cross-sectional	Buffalo, impalas, giraffe, eland, hartebeests, waterbucks	Buffalo-207, impalas-21, giraffe-1, eland-1, hartebeests-7, waterbucks-5.	4 national parks of Uganda: Murchison Falls National Park, Lake Mburo National Park, Kidepo Valley National Park, Queen Elizabeth National Park.	2005–2008.
Ayebazibwe et al. 2012.	Application of the Ceditest ^®^ FMDV type O and FMDV-NS enzyme-linked immunosorbent assays for detection of antibodies against Foot-and-mouth disease virus in selected livestock and wildlife species in Uganda.	Cross-sectional	African buffalo, cattle, goats, and sheep	218 buffalo, 758 cattle, 304 goats, and 88 sheep.	Lake Mburo, Kidepo Valley, Murchison Falls, and Queen Elizabeth National Park, a number of districts in the western and southwestern regions.	Not clearly mentioned.
Balinda et al. 2009.	Prevalence Estimates of Antibodies Toward Foot-and-mouth disease virus in small ruminants in Uganda.	Cross-sectional descriptive study	Sheep and goats	346 samples.	Bushenyi, Isingiro, Kasese, and Mbarara	2006 and 2007.
Balinda et al. 2010.	Molecular characterization of the SAT 2 foot-and-mouth disease virus from slaughtered animals after outbreak: implications for disease control in Uganda.	Cross-sectional	Cattle	12 oropharyngeal tissue samples from slaughtered cattle.	Kiboga.	2004.
Baluka 2016.	Economic effects of foot-and-mouth disease outbreaks along the cattle marketing chain in Uganda.	Qualitative and quantitative research	Cattle	873 farmers interviews, 45 cattle. KI plus 17 herds for FMD case studies.	Nakasongola, Nakaseke, Isingiro, Rakai, and Mbaale districts.	2006 to 2010.
Calkins and Scasta 2020.	Transboundary Animal Diseases (TADs) affecting domestic and wild African ungulates: African swine fever, Foot-and-mouth disease, Rift Valley fever (1996–2018).	Descriptive and retrospective	Cattle, sheep, and goats	N/A.	Countrywide.	1996 to 2018.
Dhikusooka et al. 2015.	Foot-and-mouth disease virus serotype SAT 3 in Long-Horned Ankole calf, Uganda.	Longitudinal observational	Cattle	1 positive calf.	Near Queen Elizabeth National Park.	2013.
Dhikusooka et al., 2016.	Unrecognized circulation of the SAT 1 Foot-and-mouth disease virus in cattle herds around Queen Elizabeth National Park in Uganda.	Cross-sectional	Cattle	247 sera.	Around Queen Elizabeth National Park in Uganda.	2011.
Kalema-Zikusoka et al. 2005.	A preliminary investigation of tuberculosis and other diseases in African buffalo (*Syncerus caffer*) in Queen Elizabeth National Park, Uganda.	Cross-sectional	African buffaloes	42 African buffaloes.	Queen Elizabeth National Park, Uganda.	1997.
Kasambulaet al. 2012.	Serotype Identification and VP1 Coding Sequence Analysis of Foot-and-mouth disease viruses from Outbreaks in Eastern and Northern Uganda in 2008/9.	Cross-sectional	Cattle	27 clinical samples.	Kamuli district in the eastern region and more than 10 districts in the northern region.	2008 and 2009.
Kerfua et al. 2018.	spatial and temporal distribution of foot-and-mouth disease in four districts located along the Uganda-Tanzania border: Implications for cross-border efforts in disease control.	Retrospective	Cattle	82 recorded FMD outbreaks.	Isingiro and Rakai, in Uganda, located north of the Uganda–Tanzanian border, and the two districts of Missenyi and Kyerwa, in Tanzania, located south of the Uganda–Tanzania border.	2011 and 2016.
Kerfua et al. 2019.	Low topotype diversity of recent foot-and-mouth disease virus serotypes O and A from districts located along the Uganda and Tanzania border.	Cross-sectional	Cattle	43 clinical samples.	Districts located along the Uganda and Tanzania border (Isingiro and Rakai located in the western part of Uganda, and Missoni and Kyerwa located in the northwestern part of Tanzania.	2016 and 2017.
Mugezi et al. 2020.	Risk of foot-and-mouth disease spread through cattle movements in Uganda.	Qualitative risk assessment.	Cattle	N/A.	Rakai and Isingiro.	2015 to 2019.
Muleme et al. 2012.	Effectiveness of vaccines and vaccination programs for the control of foot-and-mouth disease in Uganda, 2001–2010.	Retrospective	Livestock	Vaccination data and FMD outbreak reports from multiple districts.	Various districts in Uganda where FMD outbreaks occurred during the years 2001 to 2010.	2001 to 2010.
Munsey et al. 2019.	Spatial distribution and risk factors for Foot-and-mouth disease virus in Uganda: Opportunities for strategic surveillance.	Cross-sectional	Cattle	14,439 cattle sera from 211 herds.	7 regions of the country.	2014 to 2017.
Munsey et al. 2021.	Ecological and anthropogenic spatial gradients shape patterns of dispersal of Foot-and-mouth disease virus in Uganda.	Cross-sectional	Cattle	Not clearly defined.	Uganda, focusing on the ecological and anthropogenic factors that shape the dispersal patterns of FMDV within the country.	Not clearly mentioned.
Mwiine et al. 2010.	Serotype specificity of antibodies to Foot-and-mouth disease virus in cattle in selected districts in Uganda.	Cross-sectional	Cattle	349 cattle sera.	Bushenyi, Isingoro, Mbarara, Kasese, Mpigi, Kiboga, and Kiruhura.	2006.
Mwiine et al. 2019.	Serological and phylogenetic characterization of Foot-and-mouth disease viruses from Uganda during a cross-sectional surveillance study in cattle between 2014 and 2017.	Cross-sectional surveillance	Cattle	13,614 sera and 2,068 oral-pharyngeal fluid.	Various districts in Uganda.	2014 to 2017.
Namatovu et al. 2013.	Laboratory capacity for diagnosis of foot-and-mouth disease in Eastern Africa: implications for the progressive control pathway.	Cross-sectional	14 FMD National Referral Labs	14 FMD National Referral Labs.	Eastern Africa, covering multiple countries in the region.	2006–2010.
Namatovu et al. 2015a, b.	Challenges for serology-based characterization of Foot-and-mouth disease outbreaks in endemic areas; identification of two separate lineages of serotype O-FMdv in Uganda in 2011.	Cross-sectional	Cattle and goats	218 cattle sera, 23 goat sera, and 82 oropharyngeal fluid/epithelium tissue.	Seven Ugandan districts; Bukedea and Kumi in the eastern region, Gomba and Sembabule in the central region, Kiruhura and Isingiro in the south-west region, and Rakai in the south region.	2011.
Namatovu et al. 2015a, b.	Characterization of Foot-and-mouth disease viruses from Ugandan Cattle Outbreaks during 2012–2013: Evidence for the distribution of multiple serotypes.	Descriptive and retrospective	Cattle	79 sera samples and 60 oropharyngeal fluid/tissue/oral swab samples.	Ugandan districts, namely Kween, Rakai, Nwoya, Kiruhura, Isingiro, Ntungamo, and Wakiso.	2012–2013.
Okello et al. 2022.	Spatial and Temporal distribution of Foot-and-mouth disease in cattle in Uganda from 2010 to 2021 (A retrospective study).	Retrospective	Cattle	N/A.	Uganda 4 regions Central, Western, Eastern, and Northern.	2010–2021.
Velazquez-Salinas et al. 2020.	Genetic diversity of circulating Foot-and-mouth disease virus in Uganda cross-sectional study During 2014–2017.	Cross-sectional	Cattle	258 FMDV sequences.	29 districts representing different geographical regions in Uganda.	2014 and 2017.

The period of publication of the 36 FMD reports retrieved from the WRL-FMD and WOAH/FAO websites ranged from 1958 to 2022. The reports contained information on reported outbreaks, the type and number of samples submitted to the WRL-FMD, and the diagnostic assays used.

### Frequency of FMD outbreaks and prevalence estimates in Uganda

####  Frequency of FMD outbreaks in Uganda

Four studies reported the frequency of FMD outbreaks (Ayebazibwe et al. 2010c; Calkins and Scasta 2020; Mugezi et al. 2020; Okello et al. 2022). In the context of this study, an FMD outbreak refers to the occurrence of one or more laboratory-confirmed cases within a specific epidemiological unit such as a farm, parish, or sub-county as described in Uganda’s Risk-Based Strategic Plan for FMD control, developed by the Ministry of Agriculture, Animal Industry and Fisheries (MAAIF) (MAAIF 2021 Unpublished data). Such occurrences trigger official reporting and the implementation of control measures by veterinary authorities at the sub-county or district level, depending on the geographical extent of the outbreak. Ayebazibwe et al. (2010c) reviewed archived MAAIF data on FMD outbreaks reported by districts across the country from 2001 to 2008 and reported a range of 4–57 outbreaks and a total of 304 outbreaks during that period (Supplementary Fig. 1). Okello et al. (2022) also reviewed similar data from MAAIF for the period 2010–2021 and reported an average and median of 179 and 34 outbreaks per year, respectively, and a total of 22,690 cases of FMD in cattle within the same period, however, did not report the number of outbreaks per year. Mugezi et al. (2020) reported 140 outbreaks between 2015 and 2019 based on a review of WOAH reports. Calkins and Scasta (2020) reported 230 outbreaks following review of WOAH reports between 1996 and 2018.

Notably, there was a clear contrast between the data on annual FMD outbreaks reported in the reviewed studies and the annual reports from WRL-FMD, as illustrated in Supplementary Fig. 2. An examination of the WRL-FMD reports revealed multiple years for which data on FMD outbreaks in Uganda were missing from the annual reports published by WRL-FMD, despite Uganda’s commitments under the roadmap.

#### Prevalence estimates of FMD in cattle, small ruminants, and wildlife

A total of 12 studies reported the prevalence of FMD, with data derived from surveys or follow-up investigations of outbreak cases in which samples were collected (Supplementary Table S3). Most of the studies focused on cattle (*n* = 7) or cattle and goats (*n* = 1), and few on goats and sheep (*n* = 1), and African buffalo (*n* = 3). The findings on seroprevalence from three studies revealed that in cattle herds that had not shown recent clinical signs of FMD, the seroprevalence based on non-structural protein (NSP) ranged from 5 to 31.4% at the individual animal level and from 6 to 6.2% at the herd level (Mwiine et al. 2010; Dhikusooka et al. 2016; Munsey et al. 2019). However, when the data incorporated herds with recent or ongoing FMD cases, seroprevalence rose sharply to between 50% and 78% at the individual animal level and to 63% and 86.7% at the herd level. In small ruminants, seroprevalence was reported in only two studies and was 14% and 17% in goats (Balinda et al. 2009; Namatovu et al. 2015a, b) and 22% in sheep (Balinda et al. 2009).

In the African buffalo (*Syncerus caffer*), three studies reported seroprevalence rates of 57.1% (Kalema-Zikusoka et al. 2005), 74.2% (Ayebazibwe et al. 2010b), and 85% (Ayebazibwe et al. 2010c). In addition to African buffalo, FMD was detected using serology in other species of wildlife in a single study. Ayebazibwe et al. (2010b) investigated the role of African buffaloes in maintaining FMD in Uganda and analysed 207 serum samples from buffaloes in four major national parks. Furthermore, samples from other wildlife were analysed: 21 impalas (Aepyceros melampus), one giraffe (Giraffa camelopardalis), one common eland (*Taurotragus oryx*), seven hartebeests (*Alcelaphus buselaphus*), and five waterbucks (*Kobus ellipsiprymnus*). All samples from other species of wildlife were seronegative except one sample from hartebeest that was seropositive for FMD (Supplementary Table S3).

### FMDV serotypes and topotypes and their distribution in Uganda

The FMD serotypes were reported in 15 studies (Table 2) and 32 reports from the WRL-FMD and WOAH websites (Supplementary Table S4). Ten of the 15 studies were exclusively focused on cattle, while two studies focused on African buffalo (*Syncerus caffer)*. Furthermore, a study was conducted on both cattle and African buffalo (*Syncerus caffer)*, cattle and goats (*n* = 1) and sheep and goats (*n* = 1).

**Table 2:** The reported FMD serotypes and topotypes in different animal species in Uganda

Reference	Animal species	Serotypes serologically detected	Serotypes/topotypes isolated via culture
Ahmed et al. 2022.	Cattle.		Topotype O/EA-2 (Lineages I, II, IV, III, and IV).
*Ayebazibwe et al.*,* 2010.*	African buffalo (*Syncerus caffer*).	SAT 1 SAT 2, SAT 3.	
*Ayebazibwe et al.*,* 2010.*	Cattle and African buffalo (*Syncerus caffer*).	O, SAT 1, SAT 2 in cattle, and SAT 3 in buffalo.	SAT 1/IV, SAT 2/X.
Balinda et al. 2009.	Goats and sheep.	O, SAT 1, SAT 2, and SAT 3.	
Balinda et al. 2010.	Cattle.		SAT 2.
Dhikusooka et al. 2015.	Cattle.		SAT 3/V.
Dhikusooka et al. 2016.	Cattle.	O, SAT 1, SAT 2, SAT 3.	SAT 1/IV.
Kalema-Zikusoka et al. 2005.	African buffalo (*Syncerus caffer*).	A, O, SAT 1, SAT 2, and SAT 3.	SAT 1 and SAT 3.
Kasambula et al. 2012.	Cattle.		O/EA-2.
Kerfua et al. 2019.	Cattle.		O/EA-2 (different from EA-1 of the strain vaccine). A/Africa-G-I (different from the G-VII vaccine strain.
Mwiine et al. 2010.	Cattle.	O, SAT 1, SAT 2, and SAT 3.	
Mwiine et al. 2019.	Cattle.	A, O, SAT 1, and SAT 2.	O/EA-1 and EA-2, A/Africa G-I, SAT 1/I and IV, SAT 2/VII, IV and X.
Namatovu et al. 2015a, b.	Cattle and goats.	O, SAT 1, and SAT 3.	O/EA-2 (different from EA-1 of the vaccine).
Namatovu et al. 2015a, b.	Cattle.	O, SAT 1, SAT 2, and SAT 3.	SAT 2/I A/Africa G-I.
Velazquez-Salinas et al. 2020.	Cattle.		A/Africa G-I, O/EA-1 and EA-2, SAT 1/I and IV, SAT 2/IV, VII and X.

The studies elucidated various FMD serotypes in different animal species. In cattle, the circulating serotypes identified by isolating the virus following culture included O, A, SAT 1, and SAT 2, with serotype O being the most frequent (Kalema-Zikusoka et al. 2005; Balinda et al. 2009, 2010; Ayebazibwe et al. 2012; Kasambula et al. 2012; Namatovu et al. 2015a; Dhikusooka et al. 2015, 2016; Kerfua et al. 2019; Mwiine et al. 2019; Velazquez-Salinas et al. 2020; Ahmed et al. 2022) as shown in Table 2. This finding was consistent with the reports from WRL-FMD; the same serotypes were the most reported (Supplementary Table S4). Notably, SAT 3 serotype was isolated from a single Ankole cattle in 2013 (Dhikusooka et al. 2015). The serotypes identified using serological analysis methods included A, O, SAT 1, SAT 2, and SAT 3 (Kalema-Zikusoka et al. 2005; Balinda et al. 2009; Ayebazibwe et al. 2012; Mwiine et al. 2010, 2019; Namatovu et al. 2015a, b; Dhikusooka et al. 2016), reflecting diverse serotypes within cattle populations.

In the African buffalo (*Syncerus caffer)*, SAT serotypes were the most reported. The SAT 1, 2, and 3 serotypes were isolated using virus isolation and reported in only two studies (Ayebazibwe et al. 2010b; Kalema-Zikusoka et al. 2005). Furthermore, serological detection of antibodies to serotype O, A, SAT 1, 2 and 3 (Kalema-Zikusoka et al. 2005; Ayebazibwe et al. 2010a, 2012), was reported. In sheep and goats, serological detection identified antibodies to serotypes O, SAT 1, SAT 2, and SAT 3 (Balinda et al. 2009; Namatovu et al. 2015a, b). However, no isolation and virus characterization were performed.

**Fig. 2:**
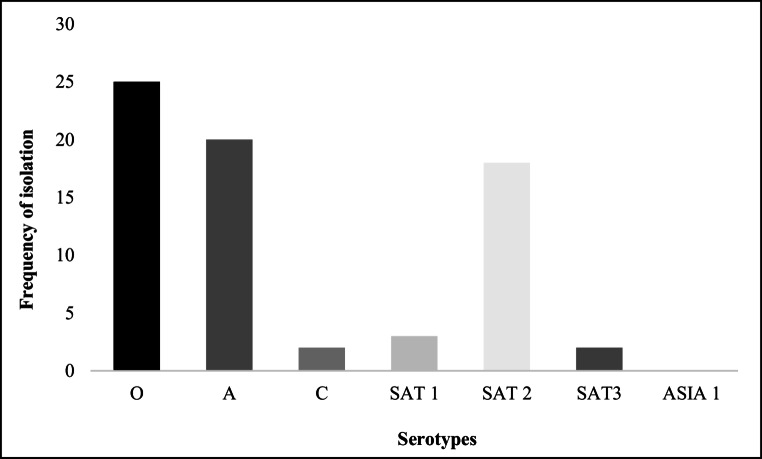
Isolated serotypes in Uganda between 1958 and 2022, based on reports from WRL-FMD and WOAH

The serotypes identified in the published studies (Table 2), corresponded to those described in the reports from the WRL-FMD quarterly and annual reports. Serotype O was the most reported (Fig. 2 and Supplementary Table S4). Additionally, the WRL-FMD report highlighted the presence of serotype C in Uganda, which is noteworthy, as it was last documented in the country in 1971 (Supplementary Table S4).

The information on the virus topotypes of isolates was reported in 10 studies for cattle (Balinda et al. 2010; Kasambula et al. 2012; Namatovu et al. 2015a, b; Dhikusooka et al. 2015, 2016; Kerfua et al. 2019; Mwiine et al. 2019; Velazquez-Salinas et al. 2020; Ahmed et al. 2022), as shown in Table 2, and 1 study in African buffalo (Ayebazibwe et al. 2010b). Within cattle populations, topotypes O/EA-1 and EA-2 were reported for serotype O, topotype A/Africa G-I was reported for serotype A, topotypes I and IV were reported for serotype SAT 1, topotypes VII, IV and X were reported for serotype SAT 2, and lastly, topotype V was reported for serotype SAT 3. The only study reporting on topotypes in the African buffalo reported the topotypes SAT 1/IV and SAT 2/X for serotypes SAT 1 and SAT 2, respectively. Another noteworthy observation was that no topotypes were reported in small ruminants.

**Fig. 3:**
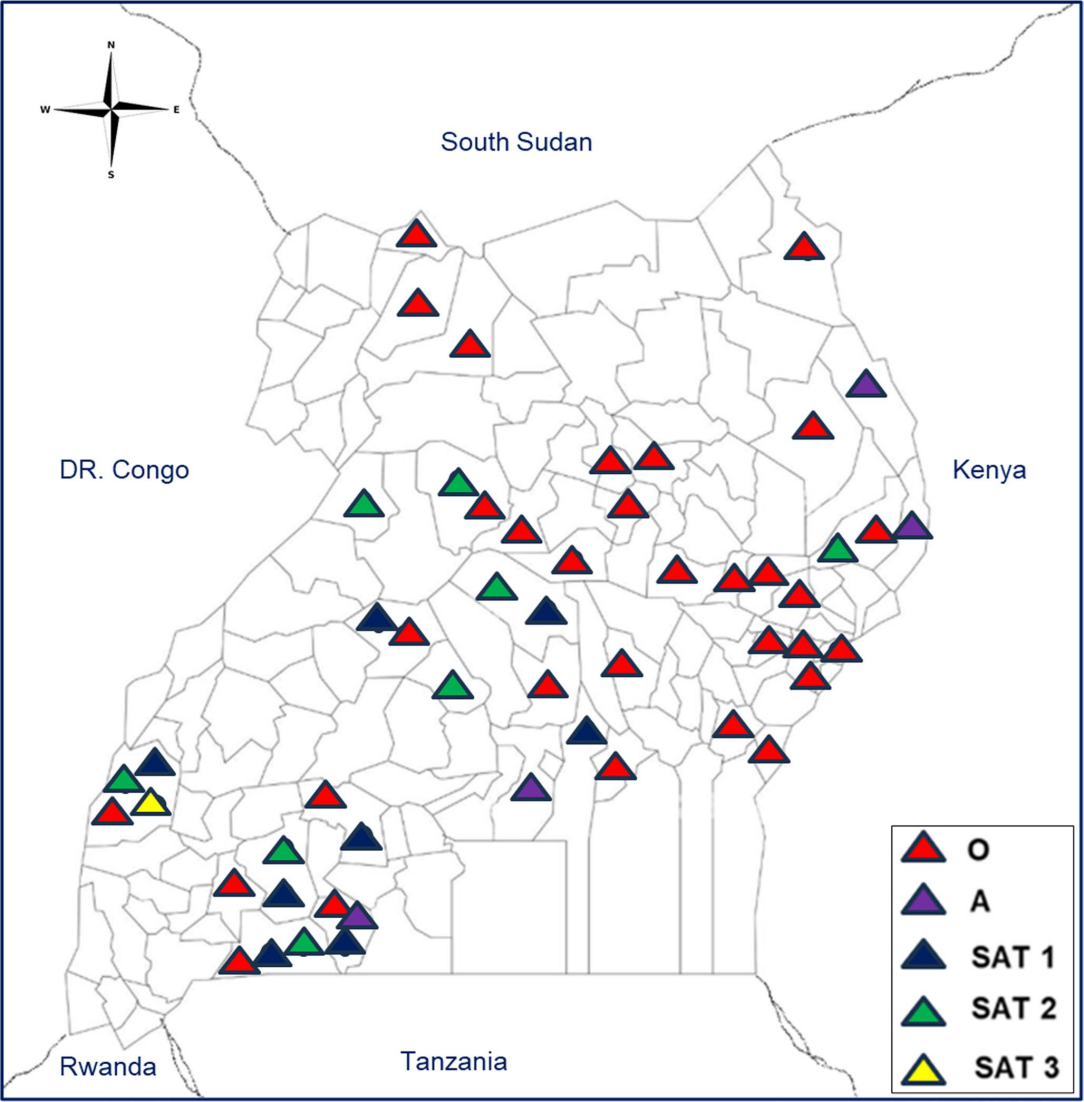
Map showing the distribution of serotypes in Uganda reported in the reviewed studies 2001–2022

The distribution of circulating serotypes is visually depicted in Fig. 3. Serotype O was the most widely distributed FMD serotype in the study districts in Uganda, followed by the SAT serotypes 1 and 2. Serotypes A and SAT 3 were notably the least widely distributed. The concentration and diversity of serotypes varied by region. For example, regions adjacent to Queen Elizabeth National Park, such as Kasese district, displayed a notable concentration of serotypes O, SAT 1, SAT 2, and SAT 3. Likewise, districts near the international border with Tanzania in the southwestern region of Uganda (Isingiro and Rakai districts) showed a pronounced distribution of serotypes O, A, SAT 1, SAT 2, and SAT 3, while the border with Kenya in the eastern part of Uganda (Nakapiriprit, Amudat, and Moroto districts) showed a marked distribution of serotypes O, A and SAT 2. The distribution of the most common circulating serotypes in various districts of Uganda was similar to that observed in the cattle corridor, an area that stretches from the southwest to the northeast regions of Uganda, covering approximately 35% of the country’s land area. This finding suggests an association between the prevalence of these serotypes and the cattle corridor area.

### Risk factors for FMD in Uganda

A total of 12 risk factors for FMD were identified in eight studies (Supplementary Fig. 3). The dry season, characterized by low average rainfall, was the most frequently reported risk factor in six of the eight studies (Ayebazibwe et al. 2010c; Kerfua et al. 2018; Mugezi et al. 2020; Munsey et al. 2019, 2021; Okello et al. 2022). Animal movements (Ayebazibwe et al. 2010c; Kasambula et al. 2012; Mugezi et al. 2020; Okello et al. 2022) and location near international borders (Kerfua et al. 2018; Mugezi et al. 2020; Munsey et al. 2019; Okello et al. 2022), were reported in four studies each. Another notable risk factor reported in three studies was pastoralism (Munsey et al. 2019; Mugezi et al. 2020; Okello et al. 2022).

High cattle density (Munsey et al. 2019, 2021), the presence of clinically sick animals (Ayebazibwe et al. 2010c; Mwiine et al. 2010), and location near national parks (Ayebazibwe et al. 2010c; Mwiine et al. 2010), were each reported by two studies. Other reported risk factors included areas located near livestock markets (Munsey et al. 2021), recent history of FMD outbreaks (Mwiine et al. 2010), communal grazing practices, purchasing cattle from farms and having young cattle aged between 12 and 24 months of age on farms (Mugezi et al. 2020).

### FMD control strategies in Uganda

The main reported FMD control measures were vaccination and quarantine. Vaccination was identified as the primary FMD control measure for reported or confirmed FMD outbreaks in five studies (Muleme et al. 2012; Namatovu et al. 2015a, b; Kerfua et al. 2019; Mugezi et al. 2020; Velazquez-Salinas et al. 2020), as shown in Supplementary Table S5. Vaccination was characterized by mostly emergency vaccinations conducted following FMD outbreaks and planned annual vaccinations targeting high risk districts. The use of quarantine was reported in 4 studies (Muleme et al. 2012; Namatovu et al. 2015a, b; Mugezi et al. 2020; Velazquez-Salinas et al. 2020). Other FMD control measures included the inspection of animals brought to market by public veterinarians (Mugezi et al. 2020), the establishment of veterinary inspection checkpoints along commonly used livestock routes (Mugezi et al. 2020) and the vigilance or disease information alertness of district veterinary officers to encourage prompt reporting of outbreaks (Namatovu et al. 2015a, b).

### FMD control challenges in Uganda

The challenges faced in controlling FMD in Uganda were highlighted in 21 studies (Supplementary Fig. 4). Limited diagnostic capacity, particularly the capacity to confirm outbreak strains, was highlighted by 6 of the 21 studies (Balinda et al. 2010; Mwiine et al. 2010; Ayebazibwe et al. 2012; Namatovu et al. 2013, 2015a, b; Kerfua et al. 2018). Virus diversity (Balinda et al. 2009; Dhikusooka et al. 2015, 2016; Ahmed et al. 2022), and vaccine mismatch (Namatovu et al. 2015a, b; Kerfua et al. 2019; Velazquez-Salinas et al. 2020) were also reported in 4 studies.

The complexities of managing inevitable and uncontrolled livestock movements, often triggered by seasonal changes, grazing practices, or trade activities, including the movement of animals across international borders, were reported by three studies (Ayebazibwe et al. 2010c; Kasambula et al. 2012; Okello et al. 2022). The presence of FMD carriers among seemingly healthy animals (Balinda et al. 2009; Dhikusooka et al. 2016), the lack of standardized FMD outbreak reporting system (Ayebazibwe et al. 2010c; Calkins and Scasta 2020), and very low national herd vaccination coverage (Kalema-Zikusoka et al. 2005; Mugezi et al. 2020), were also reported.

An additional 11 distinct challenges of FMD control were highlighted including inadequate veterinary personnel, the absence of a suitable market infrastructure for clinical examination and isolation of sick animals sold at markets, poor farm biosecurity measures, lack of quarantine facilities at animal checkpoints (Mugezi et al. 2020), lack of georeferenced data i.e. data linked to specific geographic coordinates (Ayebazibwe et al. 2010c), the potential for interspecies transmission especially at the interface between wildlife and livestock (Mwiine et al. 2019), the role of small ruminants in FMD epidemiology (Balinda et al. 2009), the use of non-purified vaccines (Ayebazibwe et al. 2012), farmers selling animal during FMD outbreaks (Balinda et al. 2010), delays in outbreak response (Muleme et al. 2012), and the ineffectiveness of quarantine procedures (Okello et al. 2022).

### Quality assessment results

The quality of the reviewed studies was assessed using the JBI Critical Appraisal Checklist, focusing on eight key criteria as shown in Supplementary Table S6. Based on the defined criteria, 15 of the studies were classified as high-quality studies, 6 as medium-quality studies, and 5 as low-quality studies. High-quality studies had clear inclusion criteria, detailed descriptions of subjects and settings, reliable measurements, and appropriate statistical analysis where applicable. Medium-quality studies generally met methodological standards but had some limitations in design, measurement, or reporting. Low-quality studies had significant methodological weaknesses, inadequate reporting, or inconsistencies in design and execution. Overall, most of the studies were of high quality, suggesting confidence in the reliability and validity of their findings. However, a subset of studies had limitations that may affect their generalizability.

## Discussion

This study aimed to review the epidemiology and control of FMD in Uganda, specifically the prevalence, circulating strains, risk factors, control strategies, and the challenges faced. The review findings revealed that most studies focused on cattle and to a limited extent African buffalo (*Syncerus caffer*) and small ruminants and no studies on swine were reported. The documented interactions between livestock (cattle, goats, sheep) and wildlife (African buffalo, antelopes, warthogs, etc.) in wildlife-livestock interface areas (Meunier et al. 2017; Byamukama et al. 2021), and the lack of studies in swine highlights the importance of understanding the role of these animal species in FMD virus transmission and persistence in the country.

A review of reports from the WRL-FMD revealed inconsistencies in sample submissions and reporting from Uganda, suggesting gaps in data collection, sharing, or communication between Uganda and the WRL-FMD. Gaps in data sharing may hinder the effective monitoring and communication of FMD updates within regional or global frameworks, as outlined in the FAO/WOAH PCP-FMD strategy for FMD control. As a participant in the FAO and WOAH-led PCP-FMD roadmap, Uganda is required to report outbreaks and submit samples to the WRL-FMD. Uganda’s current Risk-Based Strategic Plan 2021 (MAAIF RBSP– 2021) aims to address gaps in data sharing by developing a standardized national outbreak reporting framework that integrates electronic and community-based reporting systems. However, for successful implementation, farmer sensitization on the use of technology and active community engagement will be essential in fostering compliance and participation.

### Prevalence of FMD in Uganda

The findings revealed that FMD is present throughout the country and in various livestock species, including cattle, sheep, goats, and African buffalo. Animal level seroprevalence based on NSP ELISA in cattle without recent clinical signs of FMD and without history of FMD outbreaks, ranged from 5% (Mwiine et al. 2010) to 31.4% (Munsey et al. 2019) while herd-level seroprevalence ranged from 6% (Mwiine et al. 2010) to 60.2% (Munsey et al. 2019). The reported animal level prevalence may suggest subclinical or past infections, or vaccine-induced seropositivity if non-purified vaccines were used. The potential variability in interpretation of the reported prevalence hinders definitive conclusions and highlights the need for use of diagnostic techniques that can distinguish between previous infections, vaccination, and current infections. In herds with recent or ongoing FMD outbreaks, animal-level seroprevalence was higher, ranging from 50% (Namatovu et al. 2015a, b) to 78% (Mwiine et al. 2010), while herd-level seroprevalence was 63–86.7% (Munsey et al. 2019; Namatovu et al. 2015a, b). While this finding could suggest the presence of ongoing infections, it is important to consider that FMD vaccination in Uganda is often reactionary, conducted in response to an outbreak. Therefore, it is crucial to interpret seroprevalence data collected during outbreaks with caution.

In FMD-endemic settings such as Uganda, non-purified FMD vaccines are widely used in FMD control campaigns (Doel 2003), leading to challenges in interpretation of FMD test results. The use of non-purified vaccines makes it difficult to distinguish vaccinated from infected animals due to residual nonstructural proteins that may remain in traditional FMD vaccines after processing, resulting in some animals with false positive results for both SP and NSP antibodies, instead of the SP antibodies expected from purified vaccines (Hedger 1972; Mackay et al. 1998; Bergmann et al. 2000; Lee et al. 2006; Mohapatra et al. 2011). The effect of use of non-purified vaccines was reported in neighboring Kenya (Kibore et al. 2013), where an association between seropositivity and vaccination status in cattle was observed, suggesting potential misinterpretations of serosurvey data in regions where non-purified vaccines were used. In that study, the reported FMD seroprevalence in cattle was 52.5% based on NSP ELISA (Kibore et al. 2013). However, the complexities of vaccination, as described above, make country-by-country comparison of seroprevalence challenging. Similarly, differences in studies conducted in Uganda and Kenya make it difficult to make meaningful comparisons.

In small ruminants, prevalence data for FMD were limited. FMD seroprevalence of 17.7% was reported in Uganda in only two studies (Balinda et al. 2009; Namatovu et al. 2015a, b), and this was lower compared to 22.5% reported in Kenya (Chepkwony et al. 2021). The two studies used small sample sizes; 56 sheep and 143 goats in one study (Balinda et al. 2009), and 23 goats in the other study (Namatovu et al. 2015a, b). The two studies also had a narrow geographical scope; were conducted in the cattle corridor areas and did not include other areas in the country with a reported history of FMD. It is important to note that even though there is limited evidence on FMD in small ruminants in Uganda, small ruminants are susceptible to FMDV infection. A study in Nigeria recently reported severe clinical FMD in small ruminants (Olumuyiwa et al. 2023). The current study further highlights the need for robust studies to improve the understanding of FMD epidemiology in small ruminants.

In the African buffalo (*Syncerus caffer*), a prevalence of 72% was reported (Kalema-Zikusoka et al. 2005; Ayebazibwe et al. 2010a, b), and was comparable to 77% reported in Kenya (Omondi et al. 2020), 80.9% in Tanzania (Casey-Bryars 2016), and an overall prevalence of 64.8% in a pool of buffalo samples from West and Central African countries (Di Nardo et al. 2015). In the study by Di Nardo et al. (2015), prevalence varied across individual countries, ranging from 44.4% in Benin (8/18), 80.0% in Burkina Faso (4/5), 0% in Nigeria (0/1), 64.2% in the Central African Republic (52/81), 56.6% in Chad (30/53), 97.1% in the Democratic Republic of Congo (33/34), and 0% in Gabon (0/4). These prevalence figures highlight the pivotal role of the African buffalo (*Syncerus caffer*) as a reservoir for the FMD virus.

Overall, the current study revealed that multiple outbreaks occur in the country each year, ranging from 17 to 37 outbreaks per year. Annual outbreaks exhibited a consistent temporal pattern, peaking in June and July (Ayebazibwe et al. 2010c), and in November (Okello et al. 2022), aligning with periods of lower average rainfall. Similar temporal patterns have been reported in neighbouring East African countries. For example, peak outbreaks have been documented during the dry months in Tanzania (Kerfua et al. 2018), Ethiopia (Woldemariyam et al. 2023), Burundi (Garcia et al. 2022), and Rwanda (Udahemuka et al. 2020).

The observed temporal pattern of the outbreaks suggests that season is an important risk factor for FMD outbreaks in Uganda and the surrounding regions. The dry season, characterized by low average rainfall, was cited in six studies as a key risk factor for FMD (Ayebazibwe et al. 2010c; Kerfua et al. 2018; Mugezi et al. 2020; Munsey et al. 2019, 2021; Okello et al. 2022). In Uganda, the dry season often leads to a scarcity of pasture and limited water access for livestock farmers, which in turn forces farmers to move their cattle in search of shared communal grazing and watering areas. The dry season has also been reported as a risk factor for FMD in other African countries, including Ethiopia (Megersa et al. 2009; Abdela 2017), Zambia (Hamoonga et al. 2014), and South Africa (Sirdar et al. 2024). Strategies to mitigate the risk of FMD particularly during the dry season are worth considering such as sustainable water management systems, pasture planting and preservation, and regulated grazing practices. Farmer-led pasture management initiatives which leverage the available land and water sources are likely to be accepted by farmers and may help reduce livestock movements during the dry season.

### Circulating serotypes and distribution in Uganda

The study findings showed that multiple FMD serotypes exist in Uganda in different animal species, including cattle, African buffalo, sheep, and goats. In cattle, serotype O was the most isolated and reported and other isolated serotypes included A, SAT 1, SAT 2 (Kalema-Zikusoka et al. 2005; Balinda et al. 2010; Ayebazibwe et al. 2012; Kasambula et al. 2012; Namatovu et al. 2015a, b; Dhikusooka et al. 2015, 2016; Kerfua et al. 2019; Mwiine et al. 2019; Velazquez-Salinas et al. 2020; Ahmed et al. 2022), and SAT 3 (Dhikusooka et al. 2015). Serological testing in cattle detected antibodies against serotypes A, O, SAT 1, SAT 2, and SAT 3. The diverse range of FMD serotypes found in cattle presents significant challenges for FMD prevention and control. First, it is important to use caution when interpreting serotype diversity based on serological methods because of the concerns of cross-reactivity in ELISA which can potentially lead to false-positive results (Ludi et al. 2022). Second, following FMD vaccination, there is limited or no cross-protection among different FMDV serotypes and even among variant strains within the same serotype (Paton et al. 2005). This complexity is underscored by reports of mismatch between the vaccine used and the serotypes of the circulating virus. For example, a study in Uganda reported the complete absence of serotype A in the vaccine used in the country (Namatovu et al. 2015a, b), yet serotype A is one of the prevalent serotypes. Another study noted significant topotype diversity between circulating and vaccine FMD virus strains (Kerfua et al. 2019). The reported differences in serotypes and topotypes influence the choice of vaccines. Historically, a trivalent FMD vaccine has been used in Uganda, but there has been a shift towards a quadrivalent vaccine with serotypes O, A, SAT 1, and SAT 2, but with varying topotypes. In addition to the use of multivalent vaccines which can protect against multiple serotypes (Rodriguez and Grubman 2009), there is a need for continuous monitoring of circulating strains and topotypes accompanied by studies that involve matching between vaccine strains and field isolates to detect and understand possible ongoing antigenic variation and to ensure that available vaccines are effective against circulating field strains in the country.

At the regional level, the study findings align with WRL-FMD reports which indicated that Serotype O is the most widely distributed in East Africa (WRL-FMD 2023b). Uganda is found within the FMDV pool 4 (East Africa Pool) countries where serotypes O, A, SAT 1, and SAT 2 are prevalent. The observed regional trend emphasizes the importance of coordinated FMD control in the East African region. The global FMD control strategy identifies seven FMD virus pools and emphasizes the importance of regional coordination in vaccine matching and strain monitoring. However, Uganda’s current limitations, including inadequate post-vaccination monitoring and the occasional lack of virus confirmation before vaccine procurement, undermine compliance with the strategy. Strengthening laboratory infrastructure is necessary to facilitate local virus characterization, vaccine matching, and post-vaccination monitoring, ensuring that control measures align with circulating serotypes and that information is shared in a timely manner.

In the African buffalo (*Syncerus caffer*), antibodies to SAT serotypes 1, 2, and 3, and serotype O were reported. The African buffalo is a natural reservoir for SAT serotypes (Hedger and Barnett 1982; Vosloo et al. 2002a, b; Bastos et al. 2003; Vosloo and Thomson 2004; Omondi et al. 2020). Proximity to national parks increases the chances of wildlife-livestock interaction and poses a challenge for FMD control. In the present study, proximity to national parks was identified as a key risk factor for the occurrence of FMD in Uganda (Ayebazibwe et al. 2010c; Mwiine et al. 2010). o-grazing of cattle and/or small ruminants with wildlife commonly occurs in herds owned by communities located near national parks in Uganda (Rannestad et al. 2006; Meunier et al. 2017; Byamukama et al. 2021).

In sheep and goats, antibodies to serotypes O, SAT 1, SAT 2, and SAT 3 were reported in two studies. In Uganda, although cattle often share the same grazing lands as small ruminants, disease investigation and samples are rarely collected from small ruminants to test for FMD. Furthermore, unlike cattle, small ruminants are not vaccinated against FMD in Uganda. Elsewhere, the potential role of small ruminants in FMD transmission was described in Nigeria; goats on farms with FMD infected cattle were reported to exhibit severe clinical symptoms of FMD, including oral and foot lesions (Olumuyiwa et al. 2023). Laboratory investigation revealed that both goats and cattle were infected with the same FMD serotype, suggesting potential cross-species transmission (Olumuyiwa et al. 2023). Studies in Rwanda (Udahemuka et al. 2020) and Nigeria (Wungak et al. 2016) reported a higher probability of the occurrence of FMD outbreaks when sheep and goats were kept on the same farm as cattle. The limited studies on FMD in small ruminants in Uganda suggest a need for well-designed and robust studies to understand FMD epidemiology in small ruminants in Uganda.

The geographical distribution of FMD serotypes in Uganda revealed a distinct pattern, with the four main serotypes O, A, SAT 1, and SAT 2 reported in areas in the vicinity of Queen Elizabeth National Park, particularly in Kasese district. Another notable pattern was the distribution of serotypes O, A, and SAT 2 in regions along Uganda’s international borders, particularly with Tanzania (Isingiro and Rakai districts) and Kenya (Nakapiriprit, Amudat, and Moroto districts). The border regions are characterized by high animal movement and trade across borders and are therefore critical points for the transmission of FMD. In the present study, animal movements and proximity to international borders were identified as risk factors for FMD in four studies (Ayebazibwe et al. 2010c; Kasambula et al. 2012; Mugezi et al. 2020; Okello et al. 2022). Animal movement has also been associated with FMD outbreaks in Tanzania (Fèvre et al. 2006; Rweyemamu et al. 2008), the Sahel region (Duchatel et al. 2018, 2019), and multiple countries in Southeast Asia (Grace and Little 2020). Therefore, it is crucial to consider proximity to national parks and border points and animal movements when developing measures to mitigate the risk of FMD, both within the country and across borders.

The observed pattern of distribution of FMD serotypes across the ‘cattle corridor’ suggests that this area is a hotspot for FMD in Uganda. The cattle corridor extends from the southwest to the northeast across Uganda’s land area, covering approximately 35% of the country’s land area. The area has varied climatic conditions, with the west and southwest having bilateral rainfall seasons but with long dry seasons, and the north-east part being semi-arid. The cattle corridor is generally characterized by low and unreliable rainfall, prolonged droughts, and extensive pastoral rangelands (Nimusiima et al. 2013; Mayanja et al. 2020). Furthermore, the cattle corridor area holds the highest national livestock and cattle population density. High cattle density facilitates FMD spread due to close animal contact between susceptible and potentially sick animals (Munsey et al. 2019, 2021). The above characteristics of the cattle corridor are a precursor to other factors such as frequent animal movements, and shared grazing and watering areas, which are associated with the transmission of FMD.

### Control strategies for FMD in Uganda

Vaccination and quarantine were the main control strategies highlighted in five studies (Muleme et al. 2012; Namatovu et al. 2015a, b; Mugezi et al. 2020; Velazquez-Salinas et al. 2020). While vaccination was identified as a key strategy, gaps were observed in the vaccination coverage and concerns about the efficacy of the vaccine. The vaccination coverage of the national herd remains low despite FAO’s support to increase the number of FMD vaccine doses purchased from Kenya (KEVEVAPI) and Botswana (BVI) (Mwiine 2017). Analysis of national vaccination data sourced from MAAIF over a four-year period (2015/16 to 2019/20), revealed coverage of less than 5% for the 14.5 million head of cattle (Vudriko et al. 2021), and that only 1.1% of the eligible livestock were vaccinated (MAAIF 2024).

The reasons for the low vaccination coverage are not clear, but the annual procurement of insufficient number of vaccine doses may be a contributing factor, and this might be linked to budget constraints (MAAIF 2024). Budgetary constraint has been highlighted as a primary impediment restricting numerous East African countries from procuring enough doses of FMD vaccine to vaccinate a significant proportion of their national livestock populations (Hammond et al. 2021). Unlike countries with stronger economies, such as the UK, which has successfully eradicated FMD through rigorous and costly measures such as test-and-slaughter policies alongside vaccination (Lombard et al. 2007; Naranjo and Cosivi 2013), Uganda relies primarily on vaccination and quarantine, operating under budgetary and operational constraints. Relying on vaccination, along with quarantine measures and the management of clinical cases in affected cattle, may complicate control efforts by increasing the risk of creating FMD carrier cattle for up to two years after recovery (Bertram et al. 2020). Addressing these challenges and finding sustainable solutions is crucial to improving FMD control in Uganda.

Vaccination can decrease FMD virus shedding by infected animals and reduce virus transmission among vaccinated cattle populations during FMD outbreaks (Parthiban et al. 2015). Uganda has transitioned from the use of trivalent vaccines (targeting serotypes O, SAT 1, and SAT 2) to quadrivalent vaccines (covering serotypes O, A, SAT 1, and SAT 2). However, given the reported diversity of FMD serotypes and continuous evolution of the FMD virus (Longjam and Tayo 2011), routine vaccine matching and post-vaccination monitoring are necessary to ensure the effectiveness and success of the vaccination program.

The mismatch between field virus strains and vaccine strains due to the diversity of circulating viruses was reported to be a major challenge in the reviewed studies (Namatovu et al. 2015a, b; Kerfua et al. 2019). Mismatch compromises the preventive potential of vaccinations, as vaccines may not be effective against circulating strains. The FMD virus has high antigenic variability between and within serotypes, a factor that can limit cross-reactivity, and therefore the in vivo cross-protection of vaccines (Paton et al. 2005). Considering the reported multiple circulating serotypes and topotypes in Uganda, vaccine matching is crucial; however, this procedure requires well-equipped laboratories and ongoing resources, which are limited and/or lacking in the country.

Quarantine was another important FMD control strategy in Uganda highlighted in four studies (Muleme et al. 2012; Namatovu et al. 2015a, b; Mugezi et al. 2020; Velazquez-Salinas et al. 2020). The quarantine approach in Uganda typically involves implementing bans on livestock movement and trade in affected areas, within a radius of 20 km and the areas can include districts, subcounties, or parishes (MAAIF 2021 Unpublished data). The enforcement of quarantine lasts until the outbreak is fully contained with no new cases, often spanning a period of about six months (MAAIF 2021 Unpublished data). Key district authorities, such as District Veterinary Officers and support teams, Chief Administrative Officers, Resident District Commissioners, and Police, are instrumental in ensuring enforcement and adherence to quarantine measures by all stakeholders involved in the livestock movement and marketing chain (Rutebarika 2012). However, the implementation of quarantine as a control measure in Uganda comes with significant costs and challenges. Restrictions on livestock movement and trade bans can severely impact the income of farmers and traders and have broader economic repercussions due to the dependency of farmers on livestock for revenue. Consequently, these financial strains can lead farmers and traders to circumvent quarantine rules, thereby undermining the effectiveness of control measures. Non-compliance with quarantine measures may contribute to the persistence and increased frequency of FMD outbreaks. The delicate balance between disease control and livelihood preservation underscores the urgency of developing more sustainable FMD control strategies in Uganda.

### Challenges for FMD control in Uganda

The reviewed studies revealed several challenges that hinder FMD control efforts in Uganda. Multiple studies highlighted limited diagnostic capacity (Balinda et al. 2010; Ayebazibwe et al. 2010c; Mwiine et al. 2010; Namatovu et al. 2013, 2015a, b; Kerfua et al. 2018), and the reliance on external entities like WRL-FMD in the UK for FMD serotype identification. The OIE PVS Follow-Up Report (2018) for Uganda also identified limited diagnostic capacity, as well as inadequate veterinary infrastructure, and insufficient funding, as impediments to FMD control efforts. The global FMD control strategy highlights the importance of developing strong laboratory networks at both national and regional levels to enhance diagnostic capacity (WOAH/FAO 2012). At the country level, under the Risk-Based Strategic Plan (RBSP) 2026 (MAAIF 2021 Unpublished data), there is prioritization of equipping laboratories with modern diagnostic tools, training personnel in molecular diagnostic techniques, and introducing a Laboratory Information Management System to streamline operations and data management. Strengthening of these capacities will likely contribute to improved monitoring and response to FMD outbreaks.

Restricting animal movement as part of FMD control efforts in Uganda is challenging due to the continuous movement of livestock throughout the country. The continuous movements of livestock create opportunities for mixing susceptible and potentially infected animals, increasing the risk of FMD transmission (Nardo et al. 2011; Tekleghiorghis et al. 2016). In Uganda, animal movements are influenced by various factors such as seasonal changes, grazing practices, trade activities, and international border crossings (Ayebazibwe et al. 2010c; Kasambula et al. 2012; Okello et al. 2022), and rigor of enforcement of quarantine measures. Seasonal patterns, especially periods of lower rainfall are associated with the movement of livestock in search of grazing land in Uganda and neighboring East African countries (Kerfua et al. 2018; Udahemuka et al. 2020). Similarly, pastoralism and sedentary husbandry systems are commonly practiced across East Africa (Robinson et al. 2011), and are characterized by animal movements. The enforcement of quarantine measures faces challenges, including inadequate infrastructure, corruption, non-compliant farmers, nighttime animal movements, and relocations due to pasture shortages. The global FMD control strategy highlights cross-border livestock movements and wildlife interactions as major contributors to FMD spread, recommending risk-based zoning and movement controls to mitigate transmission (WOAH/FAO 2012).

The in-consistent reporting of FMD outbreaks and the absence of standardized reporting (Ayebazibwe et al. 2010c; Calkins and Scasta 2020) was another key challenge observed in the reviewed studies. Inconsistent reporting of FMD outbreaks at the national level and discrepancies in sample submission and reporting to the WRL-FMD suggest potential issues in data collection and management or communication between Ugandan authorities and WRL-FMD. Consistent and transparent data sharing is important for regional and international cooperation. Addressing these challenges can facilitate collaboration in FMD control efforts across borders and regions.

The absence of appropriate infrastructure for quarantine facilities at markets and animal checkpoints exacerbates the complexities associated with managing livestock movements in response to FMD outbreaks. Infrastructure deficiencies, including transport networks, livestock markets, and abattoirs, have been highlighted as major challenges to the control of FMD and other transboundary animal diseases in Africa (Bouslikhane 2015). Addressing the infrastructure deficiencies is necessary for improving FMD control strategies as well as enhancing the control efforts of other transboundary animal diseases in Uganda.

Overall, there is a national effort to mitigate the challenges discussed above. Uganda’s goal for FMD control under the Risk-Based Strategic Plan (RBSP) 2021–2026 is to reduce the impact of the disease on smallholder farmers by minimizing clinical cases in livestock in high-risk areas and reducing virus circulation (MAAIF 2021 Unpublished data). Since 2016, Uganda has advanced from Stage 1 to Stage 2 of the PCP-FMD (GF-TADs, 2024). However, progress has stalled at this stage. According to the RBSP draft of 2021 (MAAIF 2021 Unpublished data), Uganda aims to reach Stage 3 of the PCP-FMD by 2026, targeting a significant reduction in FMD virus circulation. The plan also sets a long-term goal of establishing FMD-free zones by 2030. Achieving these objectives will require strengthening of laboratory diagnostic capacity, expanding FMD vaccination coverage, improving surveillance systems, enforcing movement control measures during FMD outbreaks, and collaboration with livestock-keeping communities, and coordinated regional efforts. Improved FMD control in endemic countries like Uganda benefits national livestock health and productivity while reducing the risk of disease incursion into FMD-free zones globally (Jamal and Belsham 2013).

### Limitations

The following limitations were observed in the current study. The reviewed studies mainly focused on cattle and less on other susceptible livestock species such as small ruminants, pigs, and wildlife, making it difficult to fully understand the status of FMD in these species. Some of the study articles about Uganda had methodological weaknesses, specifically limited sample sizes. The limited sample size can influence the accuracy and generalizability of study outcomes. Furthermore, data gaps observed in the WRL-FMD Reports coupled with inconsistencies in reporting of outbreaks limits the ability to understand the historical and periodical overview of the FMD situation in Uganda.

## Conclusion

This review revealed that FMD remains a persistent challenge to Uganda’s livestock sector, with recurrent outbreaks. The disease continues to threaten livestock productivity, trade, and food security, with multiple serotypes circulating across different regions of the country. The findings emphasize the need for a multi-pronged approach to FMD control, including managing the high risk linked to seasonality through sustainable, farmer-led strategies such as long-term pasture planting, preservation, and water harvesting using both natural and artificial resources (rain, land and support by government and NGOs), expanding research on the role of small ruminants and wildlife in FMD transmission, improving outbreak reporting, strengthening diagnostic capacity, and implementing targeted vaccination strategies. Additionally, enhancing surveillance in high-risk areas such as border points and wildlife-livestock interfaces is crucial for early detection and containment. Strengthening these areas will be critical for Uganda to progress in the PCP-FMD pathway and achieve long-term FMD control.

## Electronic supplementary material

Below is the link to the electronic supplementary material.

Supplementary Material 1

Supplementary Material 2

Supplementary Material 3

Supplementary Material 4

Supplementary Material 5

Supplementary Material 6

Supplementary Material 7

Supplementary Material 8

Supplementary Material 9

Supplementary Material 10

## Data Availability

Data is provided within the manuscript or supplementary information files. The raw dataset supporting our findings is available on request from the corresponding author.
