# Supplementary material for: Epidemiology and control strategies for foot-and-mouth disease in livestock and wildlife in Uganda: systematic review
Source: Vet Res Commun. 2025 Jun 16;49(4):227. doi: 10.1007/s11259-025-10791-z (PMC12170765; doi:10.1007/s11259-025-10791-z)
Supplement: Supplementary file 1 — Supplementary Material 1 [file 11259_2025_10791_MOESM1_ESM.docx]

Supplementary Table S1. Description of the search terms and number of records across each of the databases*.*

| Database | Keyword combination | Records found |
| --- | --- | --- |
| PubMed | ("FOOT-AND-MOUTH DISEASE" OR "FMD") AND "UGANDA" | 52 |
|  | ("FOOT-AND-MOUTH DISEASE" OR "FMD") AND "East Africa" | 62 |
| Scopus | ("FOOT-AND-MOUTH DISEASE" OR "FMD") AND "UGANDA" | 48 |
|  | ("FOOT-AND-MOUTH DISEASE" OR "FMD") AND "East Africa" | 69 |
| Science Direct | ("FOOT-AND-MOUTH DISEASE" OR "FMD") AND "UGANDA" | 112 |
|  | ("FOOT-AND-MOUTH DISEASE" OR "FMD") AND "East Africa" | 153 |
| Web of Science | ("FOOT-AND-MOUTH DISEASE" OR "FMD") AND "UGANDA" | 39 |
|  | ("FOOT-AND-MOUTH DISEASE" OR "FMD") AND "East Africa" | 69 |
