# Supplementary material for: Epidemiology and control strategies for foot-and-mouth disease in livestock and wildlife in Uganda: systematic review
Source: Vet Res Commun. 2025 Jun 16;49(4):227. doi: 10.1007/s11259-025-10791-z (PMC12170765; doi:10.1007/s11259-025-10791-z)
Supplement: Supplementary file 2 — Supplementary Material 2 [file 11259_2025_10791_MOESM2_ESM.docx]

Supplementary Table S2. Year of publication of records retrieved from databases and the WRL-FMD and FAO/WOAH websites.

|  | **Year/period** | **Number of records** |
| --- | --- | --- |
| **FAO/WOAH web** | 1958/1991 | 1 |
|  | 1991-2000 | 9 |
|  | 2001-2005 | 5 |
| **WRL-FMD web** | 2005-2022 | 21 |
|  | **Total** | **36** |
|  | 2005 | 1 |
|  | 2009 | 1 |
|  | 2010 | 5 |
|  | 2012 | 3 |
|  | 2013 | 2 |
| **Databases** | 2015 | 3 |
|  | 2016 | 2 |
|  | 2018 | 1 |
|  | 2019 | 3 |
|  | 2020 | 3 |
|  | 2022 | 2 |
|  | **Total** | **26** |
