# Supplementary material for: Epidemiology and control strategies for foot-and-mouth disease in livestock and wildlife in Uganda: systematic review
Source: Vet Res Commun. 2025 Jun 16;49(4):227. doi: 10.1007/s11259-025-10791-z (PMC12170765; doi:10.1007/s11259-025-10791-z)
Supplement: Supplementary file 3 — Supplementary Material 3 [file 11259_2025_10791_MOESM3_ESM.docx]

**Supplementary Table S3**: The reported prevalence of FMD in different animal species.

| **Reference** | **Studies reporting FMD prevalence determined by isolation, serology, and PCR in different species** | | | | |
| --- | --- | --- | --- | --- | --- |
|  | **Cattle** | **Goats** | **Sheep** | **Buffalo** | **Hartebeest** |
|  |  |  |  |  |  |
| *Ayebazibwe et al., 2010.* |  |  |  | 74% (28/38) |  |
| *Ayebazibwe et al., 2010.* |  |  |  | 85% (175/207) | 14% (1/7) |
| *Balinda et al., 2009.* |  | 14% (20/143) | 22% (12/56) |  |  |
| *Balinda et al., 2010.* | 75% (9/12) PCR positive from post-quarantine oropharyngeal samples. |  |  |  |  |
| *Dhikusooka et al., 2015.* | 5% (1/20) Sentinel cattle were grazed near buffaloes in an area neighboring a national park. |  |  |  |  |
| *Dhikusooka et al., 2016.* | 15% (37/247) Seropositive cattle from herds around Queen Elizabeth National Park. |  |  |  |  |
| *Kalema-Zikusoka et al., 2005.* |  |  |  | 57.1% (24/42) |  |
| *Kasambula et al., 2012.* | 49% (27/55) positive on PCR testing of outbreak samples. |  |  |  |  |
| *Munsey et al., 2019.* | 37.6% (5,429/14,439) Overall individual prevalence (NSP).  56.0% Mean prevalence among herds with recent FMD (NSP).  31.4% Mean prevalence among herds with recent NO FMD (NSP).  70.6% (149/221) Overall number of positive herds (10% within herd).  86.7% (72/83) Number of herds positive with recent FMD (NSP).  60.2% (77/128) Number of herds positive without recent FMD (NSP). |  |  |  |  |
| *Mwiine et al. 2010.* | 65% (226/349) Overall individual seropositivity (NSP).  69% (239/349) Overall individual seropositivity (SP-O).  78% (222/285) Seroprevalence herds with clinical signs (NSP).  82% (235/285) Seroprevalence herds with clinical signs (SP-O).  5% (3/64) Seroprevalence herds without clinical signs (NSP).  6% (4/64) Seroprevalence herds without clinical signs (SP-O). |  |  |  |  |
| *Namatovu et al., 2015.* | 50% (109/218) Seropositive samples from outbreak investigations.  15.9% (13/82) positive samples following culture and isolation. | 17% (4/23) |  |  |  |
| *Namatovu et al., 2015.* | 77% (61/79) of NSP seropositive cattle in reported outbreaks.  63% mean seroprevalence at the herd level among the 6 herds.  26.6% (16/60) positive following culture and isolation of oropharyngeal epithelium/scrub samples. |  |  |  |  |
