# Supplementary material for: Epidemiology and control strategies for foot-and-mouth disease in livestock and wildlife in Uganda: systematic review
Source: Vet Res Commun. 2025 Jun 16;49(4):227. doi: 10.1007/s11259-025-10791-z (PMC12170765; doi:10.1007/s11259-025-10791-z)
Supplement: Supplementary file 4 — Supplementary Material 4 [file 11259_2025_10791_MOESM4_ESM.docx]

Supplementary Table S4: Data from WRL-FMD and WOAH reports showing the number of reported outbreaks, samples submitted, and serotypes isolated from 1958 to 2022.

| **YEAR** | **Number of Outbreaks** | **Samples Submitted to the WRL-FMD** | **Serotype O** | **Serotype A** | **Serotype C** | **Serotype SAT 1** | **Serotype SAT 2** | **Serotype SAT 3** | **Vaccine matching** |
| --- | --- | --- | --- | --- | --- | --- | --- | --- | --- |
| 1958 |  | 10 | + | + |  |  | + |  |  |
| 1959 |  | 69 | + | + |  |  | + |  |  |
| 1960 |  | 12 |  | + |  |  |  |  |  |
| 1961 |  | 49 | + | + |  |  | + |  |  |
| 1962 |  | 14 | + | + |  | + |  |  |  |
| 1963 |  | 19 | + | + |  |  |  |  |  |
| 1964 |  | 76 | + | + |  |  |  |  |  |
| 1965 |  | 17 | + | + |  |  |  |  |  |
| 1966 |  | 115 | + | + |  |  | + |  |  |
| 1967 |  | 49 | + | + |  |  | + |  |  |
| 1968 |  | 84 | + | + |  |  | + |  |  |
| 1969 |  | 57 | + | + |  |  | + |  |  |
| 1970 |  | 91 | + | + | + |  | + |  |  |
| 1971 |  | 87 | + | + | + |  | + |  |  |
| 1972 |  | 150 | + | + |  |  | + |  |  |
| 1973 |  | 91 | + | + |  |  | + |  |  |
| 1974 |  | 52 | + | + |  |  | + |  |  |
| 1975 |  | 60 | + | + |  |  | + |  |  |
| 1976 |  | 21 | + | + |  |  | + |  |  |
| 1977 |  | 1 |  |  |  |  |  |  |  |
| 1978 |  | 11 | + |  |  | + |  |  |  |
| 1979 |  | 0 |  |  |  |  |  |  |  |
| 1980 |  | 0 |  |  |  |  |  |  |  |
| 1981 |  | 0 |  |  |  |  |  |  |  |
| 1982 |  | 0 |  |  |  |  |  |  |  |
| 1983 |  | 0 |  |  |  |  |  |  |  |
| 1984 |  | 0 |  |  |  |  |  |  |  |
| 1985 |  | 0 |  |  |  |  |  |  |  |
| 1986 |  | 0 |  |  |  |  |  |  |  |
| 1987 |  | 0 |  |  |  |  |  |  |  |
| 1988 |  | 0 |  |  |  |  |  |  |  |
| 1989 |  | 0 |  |  |  |  |  |  |  |
| 1990 |  | 0 |  |  |  |  |  |  |  |
| 1991 |  | 0 |  |  |  |  |  |  |  |
| 1992 |  | 0 |  |  |  |  |  |  |  |
| 1993 | 6 | no info |  |  |  |  |  |  |  |
| 1994 | 12 | no info |  |  |  |  | + |  |  |
| 1995 | 15 | 4 |  |  |  |  | + |  |  |
| 1996 |  | 31 | + |  |  |  | + |  |  |
| 1997 |  | 0 |  |  |  |  |  |  |  |
| 1998 | 8 | 0 |  |  |  |  |  |  |  |
| 1999 | 0 | 10 |  |  |  | + |  |  |  |
| 2000 | 9 | 41 |  |  |  |  |  |  |  |
| 2001 | 25 | 18 | + |  |  |  |  |  |  |
| 2002 | 40 | 6 | + | + |  |  | + |  |  |
| 2003 | 72 | 0 |  |  |  |  |  |  |  |
| 2004 | 53 | 60 |  |  |  |  |  |  |  |
| 2005 | 9 | 0 |  |  |  |  |  |  |  |
| 2006 | 1 | 0 |  |  |  |  |  |  |  |
| 2007 |  | 31 | + |  |  |  |  |  |  |
| 2008 |  | 0 |  |  |  |  |  |  |  |
| 2009 | 12 | 3 |  |  |  |  |  | + |  |
| 2010 | 13 | 0 | + |  |  |  |  |  |  |
| 2011 |  | 0 |  |  |  |  |  |  |  |
| 2012 |  | 0 |  |  |  |  |  |  |  |
| 2013 | 6 | 0 |  |  |  |  |  |  |  |
| 2014 | 15 /32 | no info |  |  |  |  |  | + |  |
| 2015 |  | 0 |  |  |  |  |  |  |  |
| 2016 |  | 0 |  |  |  |  |  |  |  |
| 2017 | 9 | 2 | + |  |  |  |  |  |  |
| 2018 | 13 | no info |  |  |  |  |  |  |  |
| 2019 |  | 0 |  |  |  |  |  |  |  |
| 2020 |  | 0 |  |  |  |  |  |  |  |
| 2021 |  | 0 |  |  |  |  |  |  |  |
| 2022 |  | 0 |  |  |  |  |  |  |  |
