# Supplementary material for: Epidemiology and control strategies for foot-and-mouth disease in livestock and wildlife in Uganda: systematic review
Source: Vet Res Commun. 2025 Jun 16;49(4):227. doi: 10.1007/s11259-025-10791-z (PMC12170765; doi:10.1007/s11259-025-10791-z)
Supplement: Supplementary file 5 — Supplementary Material 5 [file 11259_2025_10791_MOESM5_ESM.docx]

**Supplementary Table S5**: Reported control strategies for FMD in Uganda.

| **Study reference** | **FMD control strategies** |
| --- | --- |
| *Kerfua et al., 2019.* | Vaccination. |
| *Mugezi et al., 2020.* | Vaccination (but vaccine coverage of 6%), quarantine, inspection of marketed animals by public vets, and veterinary inspection checkpoints along common livestock routes. |
| *Muleme et al., 2012.* | Quarantine and vaccination. |
| *Namatovu et al., 2013.* | Quarantine, vaccination. |
| *Velazquez-Salinas et al., 2020.* | Quarantine and vaccination. |
