# Supplementary material for: Epidemiology and control strategies for foot-and-mouth disease in livestock and wildlife in Uganda: systematic review
Source: Vet Res Commun. 2025 Jun 16;49(4):227. doi: 10.1007/s11259-025-10791-z (PMC12170765; doi:10.1007/s11259-025-10791-z)
Supplement: Supplementary file 6 — Supplementary Material 6 [file 11259_2025_10791_MOESM6_ESM.docx]

Supplementary Table S6. Results of quality assessment of reviewed studies performed following the Joanna Briggs Institute critical appraisal Checklist for analytical cross-sectional studies.

| **JBI Critical Appraisal Checklist for Analytical Cross-Sectional Studies** | | | | | | | | | |
| --- | --- | --- | --- | --- | --- | --- | --- | --- | --- |
| **Study Number** | **Criteria 1** | **Criteria 2** | **Criteria 3** | **Criteria 4** | **Criteria 5** | **Criteria 6** | **Criteria 7** | **Criteria 8** | **Study classification** |
|  | **Were the criteria for inclusion in the sample clearly defined?** | **Were the study subjects and the setting described in detail?** | **Was the exposure measured in a valid and reliable way?** | **Were objective, standard criteria used for measurement of the condition?** | **Were confounding factors identified?** | **Were strategies to deal with confounding factors stated?** | **Were the outcomes measured in a valid and reliable way?** | **Was appropriate statistical analysis used?** |  |
| 1 | N/A | Yes | N/A | N/A | Yes | Yes | Yes | Yes | Low |
| 2 | Yes | Yes | Yes | Yes | Yes | Yes | Yes | Yes | High |
| 3 | Yes | Yes | Yes | Yes | Yes | Yes | Yes | Yes | High |
| 4 | Unclear | Yes | N/A | N/A | Yes | Yes | Yes | Yes | Medium |
| 5 | Yes | Yes | Yes | Yes | Unclear | N/A | Yes | Yes | High |
| 6 | Unclear | Yes | Yes | Yes | Unclear | N/A | Yes | Yes | Medium |
| 7 | Yes | Yes | Yes | Yes | N/A | N/A | Yes | Yes | High |
| 8 | Yes | Yes | Yes | Yes | Yes | Yes | Yes | Yes | High |
| 9 | Yes | Yes | Yes | Unclear | Unclear | N/A | Yes | Yes | Medium |
| 10 | Yes | Yes | Yes | Yes | N/A | N/A | Yes | Yes | High |
| 11 | Yes | Yes | Yes | Yes | N/A | N/A | Yes | Yes | High |
| 12 | Yes | Yes | Yes | N/A | Yes | Yes | Yes | Yes | High |
| 13 | Unclear | Yes | Yes | N/A | Yes | Yes | Yes | Yes | Medium |
| 14 | Yes | Yes | Yes | Yes | N/A | N/A | Yes | Yes | High |
| 15 | Yes | Yes | Yes | Yes | Yes | Yes | Yes | Yes | High |
| 16 | Unclear | N/A | N/A | N/A | N/A | N/A | Yes | N/A | Low |
| 17 | N/A | Yes | N/A | N/A | Yes | Yes | Yes | Yes | Medium |
| 18 | Yes | Yes | Yes | N/A | N/A | N/A | Yes | Yes | High |
| 19 | N/A | Yes | N/A | Yes | Yes | Yes | Yes | Yes | High |
| 20 | Yes | Yes | Yes | Yes | N/A | N/A | Yes | N/A | High |
| 21 | Yes | Yes | Yes | Yes | Yes | Yes | Yes | Yes | High |
| 22 | Unclear | Unclear | N/A | N/A | N/A | N/A | Yes | N/A | Low |
| 23 | N/A | Yes | N/A | N/A | N/A | N/A | Yes | Yes | Low |
| 24 | Yes | Yes | Yes | Yes | Yes | Yes | Yes | Yes | High |
| 25 | N/A | Unclear | N/A | N/A | Unclear | N/A | Yes | Yes | Medium |
| 26 | N/A | Yes | N/A | Yes | Yes | Yes | Yes | N/A | Low |
