# Supplementary material for: Epidemiology and control strategies for foot-and-mouth disease in livestock and wildlife in Uganda: systematic review
Source: Vet Res Commun. 2025 Jun 16;49(4):227. doi: 10.1007/s11259-025-10791-z (PMC12170765; doi:10.1007/s11259-025-10791-z)
Supplement: Supplementary file 9 — Supplementary Material 9 [file 11259_2025_10791_MOESM9_ESM.docx]

**Supplementary Figure 3:** Risk factors associated with Foot-and-mouth disease in Uganda.
